# miRNA Profiles of Tubular Cells: Diagnosis of Kidney Injury

**DOI:** 10.1155/2015/465479

**Published:** 2015-04-30

**Authors:** Naoko Kito, Kosuke Endo, Masahiro Ikesue, Huachun Weng, Naoharu Iwai

**Affiliations:** Department of Genomic Medicine, National Cerebral and Cardiovascular Center, Osaka 565-8565, Japan

## Abstract

MicroRNAs (miRNAs) are small noncoding RNAs of 18–23 nucleotides that regulate gene expression. Recently, plasma miRNAs have been investigated as biomarkers for various physiological and pathological conditions. The present study details the conserved miRNA expression profiles of tubular tissues, and discusses whether they could be used to distinguish between proximal tubule injury, diagnose acute kidney injury (AKI), and the early-stage renal tubular dysfunction. miRNA expression was assessed with miRNA array and real-time reverse transcription polymerase chain reaction using the TaqMan system. The expression profiles of miR-200a/b/c, miR-145, miR-192, miR-194, miR-216a/b, miR-217, and miR-449a in human and rat tubular tissues such as the kidneys, lung, small intestine, and various exocrine glands were adequate for discriminating tubular tissues. In the kidney, miR-192 and miR-194 were highly expressed, whereas miR-145 and miR-449a were absent. miR-145 and miR-449a were relatively specifically expressed in small intestine and lung, respectively. Therefore, the combined levels of miR-200a/b/c, miR-192, and miR-194 in plasma were very useful in diagnosing AKI induced by contact freezing in mice. Moreover, urinary miR-200a levels were useful for the diagnosis of renal tubular dysfunction in Dahl salt-sensitive rat with high salt administration. Our results indicate that miRNA expression profiles are useful as biomarkers for identification of various kidney injuries.

## 1. Introduction

MicroRNAs (miRNAs) are small noncoding RNAs, comprising approximately 18–23 nucleotides, which bind to the 3′-untranslated region of messenger RNAs to repress translation or promote degradation [1, 2]. miRNA profiles reflect various physiological and pathological conditions [3, 4]. They are expressed in a tissue- or cell-specific manner [5]. The expression levels of miRNAs change in accordance with the various physiological processes [6, 7], and most of the human protein-coding genes are thought to be targeted by miRNAs [8, 9]. The distributions of major miRNAs are highly conserved across species, and miRNAs are thought to have potential as clinically relevant biomarkers. Recently, more than 2000 human miRNAs have been identified (http://www.mirbase.org/). The presence of miRNAs in various body fluids has been reported [8, 10, 11], and the use of these miRNAs as biomarkers for tissue injury has been attempted. Plasma miRNAs are contained into the RNA-induced silencing complex and into exosomes and/or microparticles [12–14]. We have previously reported the use of plasma miRNAs for determining myocardial infarction [12, 14] and cerebral infarction [15].

In the present study, we searched for conserved miRNA profiles in tissues with a similar structure and for miRNAs that could discriminate the proximal tubule. The proximal tubule, part of the duct system of the nephron in the kidney, has a tubular structure as its name suggests. In addition, the kidney has other regions with a tubular structure: the distal tubule and collecting duct. The blood vessels and intestines also have tubular structures. Other tissues, including the lung, liver, and exocrine glands, have segments with a tubular structure. The lung has bronchial tubes and pulmonary alveoli. The liver and exocrine pancreas share a common structure, with functioning units connected to the ductal tree [16], and the structure of the salivary gland closely resembles a pancreas. Thus, we determined the miRNA profiles of tissues with a tubular structure and assessed their ability to discriminate tissues with a tubular structure. We also assessed whether the expression profiles were useful for the diagnosis of kidney injury.

## 2. Materials and Methods

### 2.1. Total RNA from Human Samples

Human RNAs from various tissues (pancreas, salivary gland, prostate, lung, trachea, kidney, colon, spleen, liver, brain, heart, and skeletal muscle) were obtained from Clontech-Takara (CA, USA) and total RNAs from cell samples (proximal tubule and mesangial cells) were obtained from ScienCell Research Laboratories (CA, USA).

### 2.2. Total RNA from Rat Samples

Tissues (kidney, pancreas, salivary gland, prostate, lacrimal gland, lung, bile duct, liver, heart, skeletal muscle, spleen, and small intestine) were harvested from male and female SD rats (4 weeks old) purchased from Japan SLC (Shizuoka, Japan). Total RNA was extracted from rat tissues with TRIzol reagent (Invitrogen, CA, USA) as described previously [17]. To assess cell-specific miRNA expression in rats, the trachea, mammary gland, small intestine, and kidney were removed and immediately immersed in dry ice/ethanol with optimal cutting temperature compound and stored at −80°C. Samples were sectioned at 6 *µ*m and mounted on polyethylene naphthalate membrane slides for microdissection on a cryostat at −20°C. Slides were fixed by immersion in 95% ethanol for 30 s. Cresyl violet and eosin Y solutions were mixed and directly applied to the sections. The sections were incubated for 20–30 s, rinsed twice (1 min each) in 95% ethanol, dehydrated in 100% ethanol twice (1 min each), and washed twice (9 min each) with xylene to remove the alcohol. Then, the bronchial gland, mammary gland, intestinal villus, proximal tubule, and glomerulus were isolated from tissue sections using laser microdissection (LMD, Leica LMD 7000; Leica Microsystems, Germany). Samples were collected into the 1.05× denaturing solution from the mirVana PARIS Kit (Ambion, TX, USA), mounted onto the collection device, and purified according to the manufacturer's instruction [17].

### 2.3. miRNA Array Analysis

Expression profiling of miRNAs was performed using the ABI TaqMan MicroRNA Array kit (Applied Biosystems, CA, USA), according to the manufacturer's instructions. As an endogenous control, U6 small nuclear RNA, included in the TaqMan MicroRNA Array, was used. The ABI Prism 7900 HT Sequence Detection System (Applied Biosystems) was used for amplification and detection. *C*_*P*_ values were obtained from the amplification plot using the SDS software and RQ Manager (Applied Biosystems), as described previously [18]. The miRNA array data and the array definition have been submitted to Gene Expression Omnibus (http://www.ncbi.nlm.nih.gov/geo/) with accession number GSE66134 for human samples and GSE66195 for rat samples. *C*_*P*_ values of >35 were considered below the detection level of the assay; therefore, only miRNAs with a *C*_*P*_ of ≤35 were included in the analysis. The clustering analysis was performed using Cluster 3.0 [19] and Java Treeview [20] software programs.

### 2.4. Validation of miRNA Expression in Tissue RNA

The miRNA array system was used as a screening tool, and the results were validated with real-time reverse transcriptase-polymerase chain reaction (RT-PCR) using a TaqMan microRNA RT-PCR kit (Applied Biosystems). The real-time RT-PCR was performed using a 7500 Fast Real-Time PCR System (Applied Biosystems). *C*_*P*_ values were obtained from the amplification plot using SDS software (Applied Biosystems). In addition, to investigate the reliabilities of commercially available kits, real-time RT-PCR was performed using reference samples containing known amounts of synthetic miRNA. The concentrations of miR-200a/b/c were estimated based on the analytical curve for synthetic miRNA.

### 2.5. Assessment of Plasma miRNAs as Biomarkers for Acute Kidney Injury and Renal Tubular Dysfunction

Acute kidney injury (AKI) was induced in mice by contact freezing the kidney surface with dry ice as described previously [21]. Male C57BL/6J mice (8 weeks old) were purchased from SLC Japan (Shizuoka, Japan). The mice were maintained in a temperature-controlled room with a 12 h light/12 h dark cycle and were fed standard mouse chow (Oriental Yeast, Tokyo, Japan) and tap water ad libitium. Venous blood samples were collected into ethylenediaminetetraacetic acid- (EDTA-) containing vials at 6 h after the contact freezing or sham operation (*N* = 8 per group). Plasma was isolated by centrifugation at 1600 ×g for 15 min at 4°C. Plasma RNA was isolated using the mirVana PARIS kit (Ambion). As an internal reference, a known amount of synthetic miRNA was added, as described previously [12, 17, 22].

The Dahl salt-sensitive (DS) rat was used as renal tubular dysfunction model. A high-salt diet increases blood pressure, urinary protein excretion, and renal interstitial fibrosis in DS rats [23]. Moreover, macrophage infiltration, T-cell infiltration, and urinary albumin excretion, namely, renal tubular dysfunction, are induced within a week after administration. DS rats (4 weeks old) were purchased from SLC Japan. The rats were maintained in a temperature-controlled room with a 12 h light/12 h dark cycle. They were fed a low- (control group, *N* = 6: 0.03%) or high- (kidney injury group, *N* = 6: 8%) salt rat diet (Oriental Yeast) and tap water ad libitum. The spot urine was collected at days 0, 1, 3, 6, and 10. Urinary RNA was isolated using the mirVana PARIS kit (Ambion). As an internal reference, a known amount of synthetic miRNA was added to the urine samples. Urinary albumin and creatinine were measured by Monolis Co., Ltd. (Tokyo, Japan). After 10 days of treatment, the rats were sacrificed, venous blood samples were collected into EDTA-containing vials for miRNA measurements, and the kidneys were resected for histological examination.

The present study was conducted in accordance with the guidelines of the National Cerebral and Cardiovascular Center for the care and use of experimental animals and the National Institutes of Health Guide for the Care and Use of Laboratory Animals. Adequate measures were taken to minimize the animals' pain and discomfort.

### 2.6. Histological Examination

Kidney tissue was fixed in 10% formaldehyde and embedded in paraffin. Sections were prepared and stained with hematoxylin and eosin and Masson's trichrome. Tubulointerstitial injury in the renal cortex was assessed according to the percentage area of fibrosis, estimated using Masson's trichrome-stained sections. For each kidney, 10 microscopic fields (×400 magnification) were randomly chosen under a fluorescence microscope (Nikon, Japan), and the area of renal fibrosis was measured and analyzed using analysis software (ImageJ; National Institutes of Health, MD, USA).

### 2.7. Statistical Analysis

Data were presented as the mean ± SD. For statistical analysis, analysis of variance, regression analysis, and contingency table analysis were performed using the JMP statistical analysis package (SAS Institute, NC, USA).

## 3. Results

### 3.1. miRNA in Tissues with Tubular Structures

**Figure 1:**
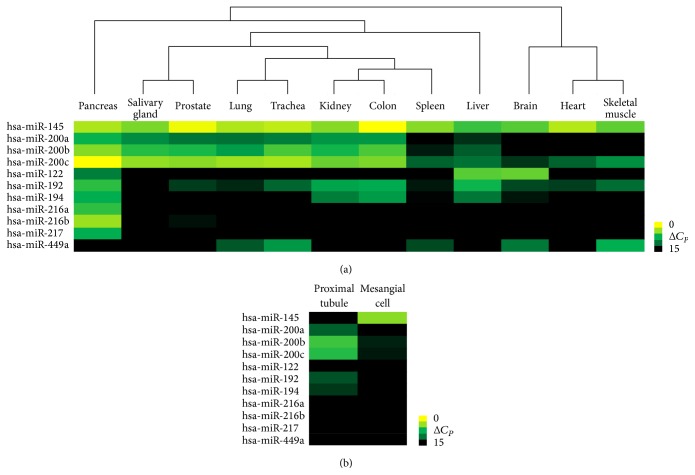
Heat map of microRNA (miRNA) expression in human tissues. (a) The expression profiles of 377 miRNAs in human tissues were examined with miRNA array analysis. Cluster analysis was performed with miR-122, miR-145, miR192, miR-194, miR-216a, the miR-200 family members, miR-217, and miR-449a. miR-200a, miR-200b, and miR-200c were highly expressed in the kidney, salivary gland, prostate, and colon. In addition, miR-200b and miR-200c were expressed in the lung, pancreas, and trachea. The cluster analysis discriminated between tubular tissues and tissues without a tubular structure (brain, heart, and skeletal muscle). Moreover, the lung and trachea were classified into the same branch. (b) The expression profiles of the 377 miRNAs in the human proximal tubule and mesangial cells were compared. Members of the miR-200 family were highly expressed in the proximal tubule.

In human tissues, the results of the miRNA array analysis showed that miR-200a, miR-200b, and miR-200c (miR-200 family) were highly expressed in the kidney, lung, salivary gland, trachea, colon, prostate, liver, and pancreas (Figure 1). To examine the miR-200 family in the kidney, miRNA array analysis was performed to compare expression in the proximal tubule and mesangial cells. Members of the miR-200 family were highly expressed in the proximal tubule.

**Figure 2:**
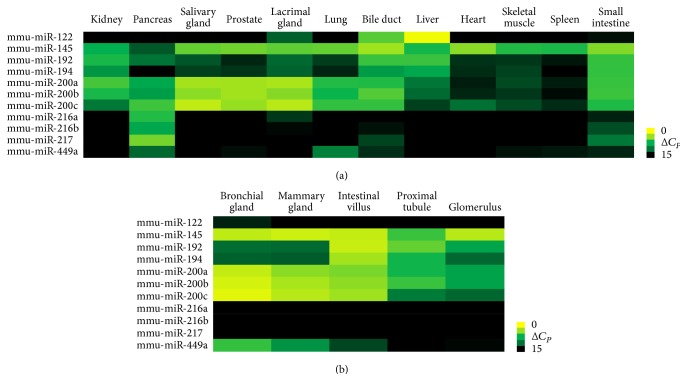
Heat map of miRNA expression in rat tissues. (a) The expression profiles of 641 miRNAs in rat tissues were examined with miRNA array analysis. miR-200a, miR-200b, and miR-200c were highly expressed in the kidney, pancreas, salivary gland, prostate, lacrimal gland, lung, bile duct, and small intestine. Moreover, miR-122, miR-145, miR192, miR-194, miR-216a, the miR-200 family members, miR-217, and miR-449a exhibited a characteristic expression pattern in each tissue. (b) Small tissues consisting of epithelial cells were collected by laser-capture microdissection, and the miRNA expression profiles were examined. miR-200a, miR-200b, and miR-200c were highly expressed in these tissues. When the proximal tubule and glomerulus were compared, the expression of miR-200a, miR-200b, and miR-200c was higher in the proximal tubule than in the glomerulus.

To investigate the distributions of the miR-200 family and identify combinations of miRNAs for tissue discrimination, miRNA array analysis was performed using rat tissues, including the kidney, pancreas, bile duct, salivary gland, prostate, lacrimal gland, lung, liver, heart, skeletal muscle, spleen, and small intestine. Members of the miR-200 family were highly expressed in the kidney, lung, small intestine, and exocrine glands (Figure 2(a)). The distributions of the miR-200 family members were investigated using an LMD system. miRNA analysis was used to assess miRNA expression in the tissue compartments, including the bronchial gland, mammary gland, intestinal villus, renal tubular cells, and glomerulus (Figure 2(b)). As expected, members of the miR-200 family were highly expressed in exocrine glands and epithelial cells. Furthermore, the levels of miR-200a/b/c were higher in the proximal tubule than in the glomerulus.

**Figure 3:**
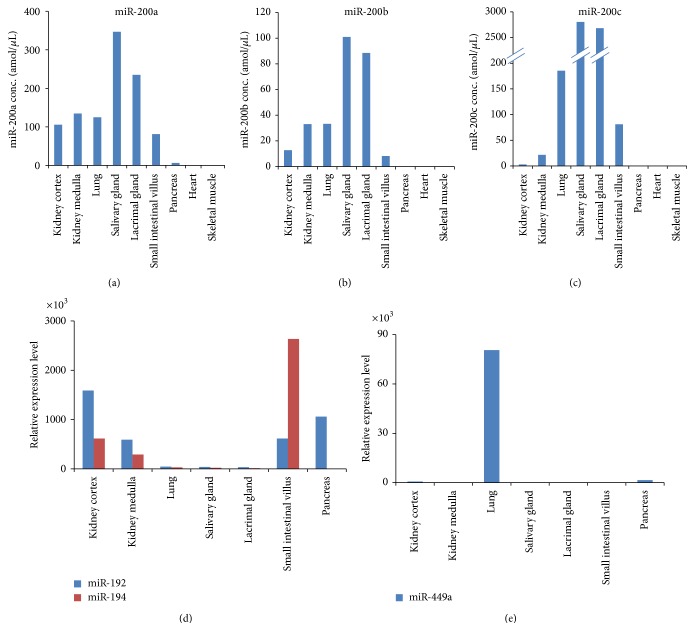
Validation of miR-200a, miR-200b, and miR-200c expression in rat tissues. The expression of (a) miR-200a, (b) miR-200b, and (c) miR-200c in rat tissues was validated with real-time reverse transcriptase-polymerase chain reaction (RT-PCR). High expression of the miR-200 family members was observed in each tissue. miR-200c was highly expressed in the lacrimal gland and salivary gland. (d) miR-192, miR-194, and (e) miR-449a were also included in the validation process. miR-192 and miR-194 were highly expressed in the kidney and small intestine, and miR-449a was highly expressed in the lung.

The expression of miR-200a, miR-200b, miR-200c, miR-192, miR-194, and miR-449a was validated with real-time RT-PCR in rat tissues in order to discriminate the kidney from other tissues with a tubular structure. Members of the miR-200 family were expressed at high levels in each tissue with a tubular structure (Figures 3(a)–3(c)). In particular, they were highly expressed in the lacrimal gland and salivary gland. On the other hand, miR-192 and miR-194 were highly expressed in the kidney and small intestine, and miR-449a was highly expressed in the lung (Figures 3(d) and 3(e)).

To confirm that a combination of miRNAs could discriminate tubular tissues, clustering analysis was performed with human samples (Figure 1). Tubular tissues were clearly discriminated from tissues without a tubular structure (brain, heart, and skeletal muscle). Furthermore, classification of the lung and trachea into the same branch showed histological consistency.

### 3.2. Assessment of Plasma miRNAs as Biomarkers of Acute Kidney Injury in Rodent Models

**Figure 4:**
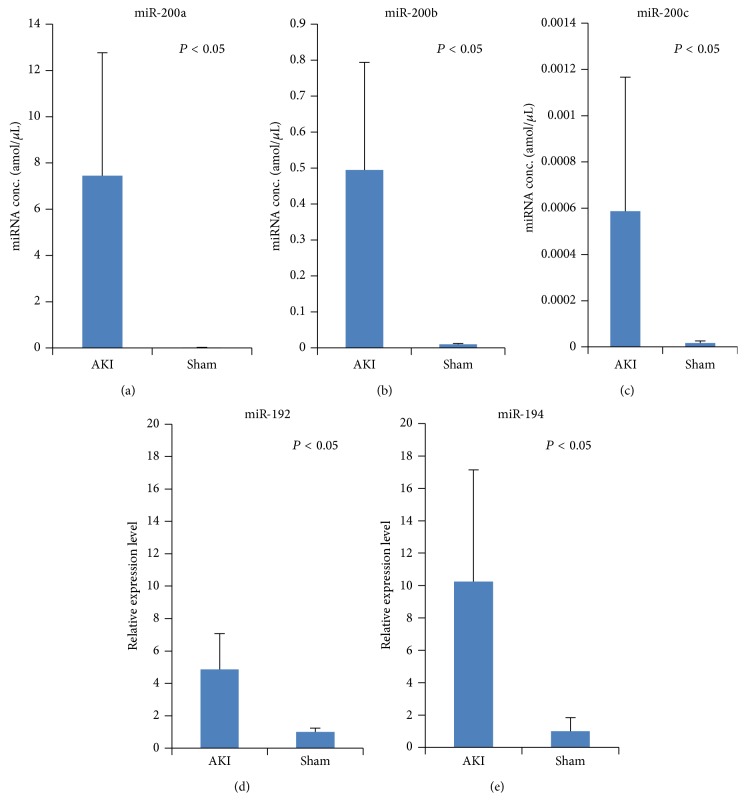
Assessment of plasma miR-200a, miR-200b, and miR-200c in a mouse model of acute kidney injury (AKI). The concentrations of (a) miR-200a, (b) miR-200b, (c) miR-200c, (d) miR-192, and (e) miR-194 were measured with real-time RT-PCR 6 h after AKI was induced by contact freezing with dry ice. All miRNAs were significantly increased in the AKI model. The columns with bars represent the mean ± SD. ^*∗*^ *P* < 0.05.

We assessed whether the plasma concentrations of miR-200a, miR-200b, and miR-200c could be used as a biomarker for acute kidney injury (Figure 4). AKI was induced in mice by contact freezing with dry ice. Visual inspection at sacrifice confirmed the induction of necrosis in the cortex. A significant increase in plasma miR-200a/b/c, miR-192, and miR-194 levels was observed in the AKI model.

### 3.3. Assessment of Urinary miRNAs as Biomarkers for the Early Stage of Chronic Kidney Disease in Rodent Models

**Figure 5:**
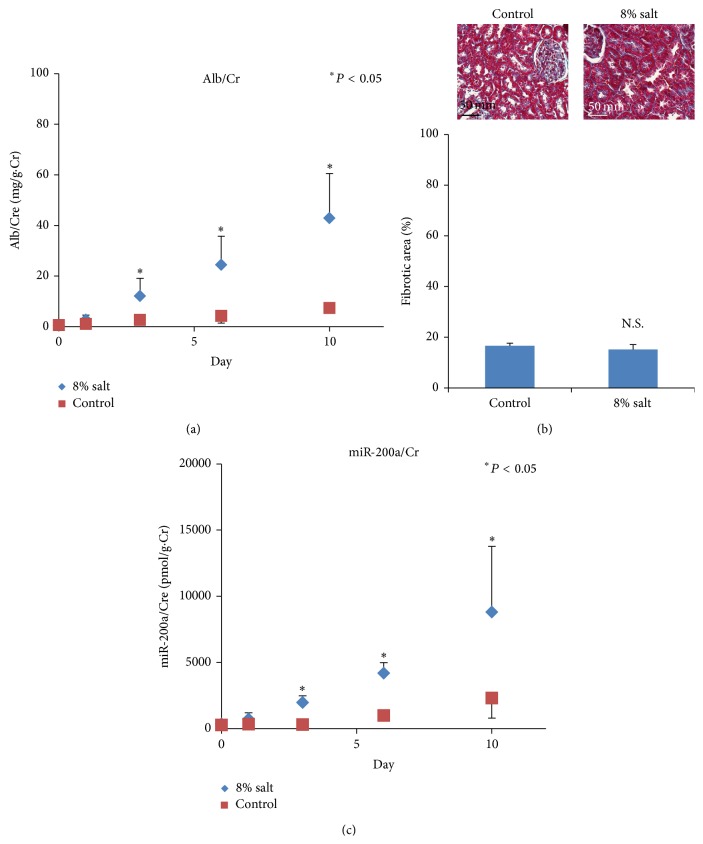
Assessment of urinary miR-200a in renal tubular dysfunction in rats. Urinary miRNA concentrations were measured with real-time RT-PCR 0, 1, 3, 6, and 10 days after administration of an 8% salt diet. (a) A high-salt diet induced urinary albumin excretion after day 3 of administration. (b) Light micrographs (Masson trichrome staining) of the kidney cortex at day 10. No significant difference in the percentage area of fibrosis was observed. N.S.: not significant. (c) Urinary miR-200a increased after day 3 of administration. The plot with bars shows the mean ± SD. ^*∗*^ *P* < 0.05.

We assessed whether the urinary concentrations of miR-200a and miR-200c could be used as a biomarker for renal tubular dysfunction (Figure 5). A high-salt diet induced urinary albumin excretion after day 3 of administration, but fibrosis was not observed (Figures 5(a) and 5(b)). Plasma miR-200a was not detected (data not shown). On the other hand, urinary miR-200a increased after day 3 of administration (Figure 5(c)). miR-200c was not detected in urine.

## 4. Discussion

Members of the miR-200 family are highly expressed in the kidney and lung. Our results showed that miR-200 family members were expressed at high levels in various tissues with a tubular structure: the kidney (proximal tubule and collecting duct), lung, pancreas (duct cells), small intestine (intestinal villus), bile duct, and exocrine glands (duct cells). These results suggest that the miR-200 family is closely associated with tubular structure. In multicellular organisms, epithelial tubular tissues are essential structures in many organs, including the lung, gut, kidney, and exocrine glands. Regulation of renal development by miRNAs is a developing field of research [24]. Patel et al. have reported that miR-200 family members play important roles in renal tubule maturation by repressing* Pkd1* in the kidney [25]. The 3′-UTR of* Pkd1* contains two evolutionarily conserved miR-200b/c binding sites. Downregulation of miR-200 family members might underlie kidney cyst formation in Dicer mutant kidneys. Furthermore, the decreased expression of miR-192 seems to correlate directly with tubulointerstitial fibrosis and a low glomerular filtration rate, and* TGF-β* suppresses miR-192 expression in cultured proximal tubular cells [26]. The miR-200 family consists of five members (miR-200a, miR-200b, miR-200c, miR-141, and miR-429), which are clustered and expressed as the miR-200b-200a-429 cluster at chromosomal location 1p36 and the miR-200c-141 cluster at chromosomal location 12p13. It has been suggested that different cell lines regulate miR-200 expression through distinct epigenetic mechanisms [27]. In the present study, the expression of miR-200c was higher in the lacrimal gland and salivary gland than in other tubular tissues. The lacrimal gland and salivary gland are formed from the ectoderm, and the expression ratio of miR-200a/b and miR-200c might depend on the germ layer.

Members of the miR-200 family are commonly expressed in tubular tissues, and it is impossible to classify these tissues using only the miR-200 family. Thus, in the present study, we characterized tubular tissues by measuring the expression of other miRNAs. Several tissues with a tubular structure were easily discriminated. miR-122 is a well-known liver-specific miRNA [11], and a number of studies have reported the usefulness of miR-122 as a biomarker for liver injury [28, 29]. Indeed, miR-122 was highly expressed in the liver and bile duct in our study. Previous studies have reported that miR-216a, miR-216b, and miR-217 are specifically expressed in the pancreas; these miRNAs were useful as biomarkers for pancreatic injury [30]. miR-192 and miR-194 were highly expressed in the kidney and in the small intestine. We previously described the miRNA profiles of muscle tissues and reported that the plasma concentration of miR-145 was increased in ischemic colitis [18]. Therefore, the absence of miR-145 and other muscle-specific miRNAs, that is, miR-133a and miR-133b, might clarify kidney injury. In addition, we identified miR-449a as a lung-specific miRNA in rodents in the present study. Furthermore, the results of the clustering analysis of human samples showed that an appropriate combination of miRNAs could discriminate tissues with a tubular structure. Although the human samples used in present study were pooled from varying numbers of individuals of random age and sex, all samples came from Caucasians (see GEO accession number GSE66134). Therefore, an effect of race is possible. However, the miRNAs identified in this study showed similar expression patterns in humans and rats. Thus, the expression of these miRNAs appears to be conserved across species, indicating that the effect of race can be excluded.

We assessed whether the miR-200 family could be used as a biomarker for kidney injury. In AKI, proximal tubules exhibit severe necrosis, and miRNAs leak into the blood. Consistently, the plasma concentrations of the miR-200 family members and miR-192 and miR-194 increased significantly. On the other hand, injury to proximal tubules progresses slowly in renal tubular dysfunction. Although morphological changes were not observed and miR-200a was not detected in plasma, macrophage infiltration was induced [23]. In the case of the renal tubular dysfunction, an increase in urinary albumin excretion was used clinically for diagnosis. In our results, urinary miR-200a also increased, and the sensitivity was similar to that of urinary albumin excretion. In the future, we will assess the potential of the urinary miR-200 family as biomarkers of the renal tubular dysfunction in humans.

## 5. Conclusion

We found that a combination of miRNAs was useful for the discrimination of tissues with a tubular structure. Our results suggest that the expression pattern of tubular tissue-specific miRNAs can accurately distinguish various diseases.
